# Hypertrophic Osteoarthropathy Associated with Probable Smear-Negative Pulmonary Tuberculosis

**DOI:** 10.1155/2022/5429138

**Published:** 2022-08-05

**Authors:** Mohamed Ahmed Ghassem, Abdelhamid Biyi, Julien H Djossou, Toufik Hamza, Abderrahim Majjad, Lahsen Achemlal

**Affiliations:** ^1^Rheumatology Department, Military Hospital Mohammed V, Mohammed V University, Rabat, Morocco; ^2^Department of Internal Medicine, National Hospital Centre, Nouakchott, Mauritania; ^3^Department of Nuclear Medicine, Military Hospital Mohammed V, Mohammed V University of Rabat, Rabat, Morocco

## Abstract

Association of hypertrophic osteoarthropathy (HOA) with pulmonary tuberculosis is rarely reported, especially with smear-negative pulmonary tuberculosis (SNPT), in which its diagnosis is a challenge. We used a systematic approach to analyze all relevant literature reviews, and we identified only two cases of HOA associated with pulmonary tuberculosis in the last 10 years. We report the case of a 36-year-old man who presented with bilateral symmetric polyarthralgia and digital clubbing. Laboratory exams associated elevated acute phase reactants with negative immunological examinations. Two series of three acid-fast *Bacillus* (AFB) smear microscopy in sputum, separated by 15 days of broad-spectrum antibiotic therapy, were negative. A sputum culture was negative for *Mycobacterium tuberculosis*. A chest X-ray and computed tomography (CT) showed an apical pulmonary cavity. Plain X-ray and bone scintigraphy revealed periostosis of the tubular bones. Therefore, the diagnosis of HOA associated with probable SNPT was made. HOA symptoms had remitted after 3 months of antitubercular therapy. After 7 months of treatment, chest CT and bone scintigraphy showed a regression of the pulmonary cavity and disappearance of periostosis. The search for tuberculosis in front of any HOA seems to be justified in our epidemiological context.

## 1. Introduction

Hypertrophic osteoarthropathy (HOA) is a clinicoradiological syndrome classically secondary to neoplastic diseases (paraneoplastic syndrome) [1]. It is most often secondary to pulmonary neoplasms (80% of cases) [2]. The association of HOA with pulmonary tuberculosis is classic but remains rare [3, 4]. Therefore, we report a case of HOA associated with probable smear-negative pulmonary tuberculosis (SNPT).

## 2. Case Presentation

A 36-year-old man was admitted to our department for bilateral symmetric polyarthralgia touching large and small joints for two months. He also complained of bone pain in the forearm and legs, shortness of breath, and dry cough without dyspnea. He was not a smoker or alcoholic. His medical history was without specialties, including lung and liver diseases.

**Figure 1:**
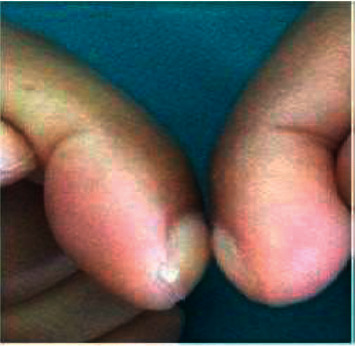
Digital clubbing and positive Schamroth's sign.

On physical examination, vital signs, including body mass index, were normal. A musculoskeletal examination revealed a digital clubbing with Schamroth's sign (Figure 1), bilateral and symmetric swelling of the elbow, wrist, and ankles. Palpation of the bony reliefs of the forearms and legs is also painful. The rest of the somatic examinations were normal including the lungs, heart, liver, and skin.

Laboratory findings revealed elevated acute phase reactants with erythrocyte sedimentation rate (ESR) at 90 mm and C-reactive protein (CRP) at 98.1 mg/L. Immunological examinations were negative (antinuclear antibodies (ANAs), anticitrullinated protein antibodies (ACPAs), rheumatoid factors (RFs), antinuclear antibodies (ANAs), and anti-ds DNA antibodies). Serum thyroid-stimulating hormone (TSH) and free thyroid hormones (FT3 and FT4) were normal. Renal and hepatic functions were within the normal range. Acid-fast *Bacillus*(AFB) smear microscopy in sputum samples of 3 successive days was negative. A sputum culture was negative for *Mycobacterium tuberculosis*. Bronchoscopy was normal and cultures from bronchoalveolar lavage (BAL) samples were negative for Mycobacteria and Aspergillus. Hydatid and Aspergillus serologies were negative. The human immunodeficiency virus antibody test was negative.

**Figure 2:**
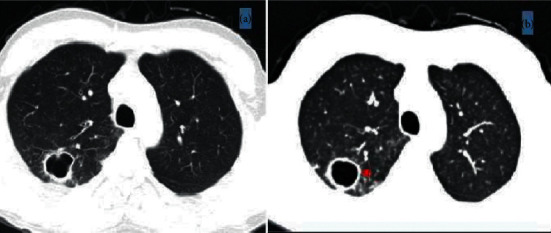
(a) Chest CT showing an apical pulmonary cavity in the right lung with bronchiolitis and hilar lymphadenopathy. (b) The chest CT performed 8 months later showing a regression of the pulmonary cavity (arrowhead).

**Figure 3:**
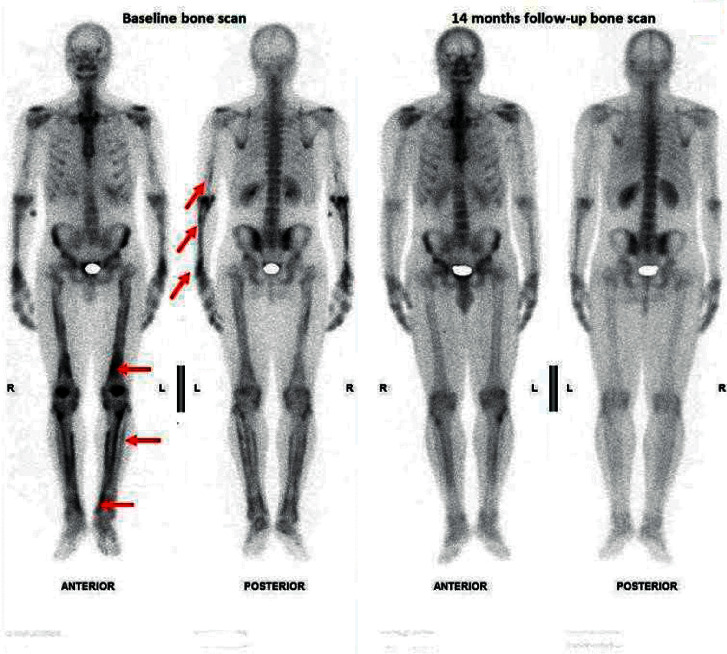
(a) Bone scan showing increased radiotracer uptake (arrow) along the tubular bones of the upper and lower extremities consisting with periostosis. (b) The whole-body scan performed 14 months later showing the improvement of the scintigraphic abnormalities.

A chest X-ray and computed tomography (CT) showed an apical pulmonary cavity in the right lung with bronchiolitis and hilar lymphadenopathy (Figure 2(a)). Plain X-ray revealed a monolayer periosteal reaction along the bones of the forearm and legs without acro-osteolysis. Bone scintigraphy showed longitudinally radiotracer uptake along all long bones of the upper and lower extremities confirm periostosis of the tubular bones (Figure 3(a)).

In front of digital clubbing, we discussed acromegaly, thyroid acropachy, and HOA. However, the absence of prognathism and the presence of periostosis quickly excluded acromegaly. Thyroid acropachy is a manifestation of Graves' disease, in our patient, eliminated by the absence of Graves' ophthalmopathy and pretibial myxedema with TSH normal. The negativity of immunological examinations excluded inflammatory arthritis. The diagnosis of HOA was made based on the association of digital clubbing, periostosis of the tubular bones, and seronegative arthritis. Given the negativity of the AFS smear microscopy and the presence of an apical pulmonary cavity in the right lung, in our epidemiological context of tuberculosis, the diagnosis of SNPT was presumed. Indeed, our patient was treated with broad-spectrum antibiotics for 15 days but without clinical improvement. The second series of 3 AFS smear microscopy in sputum samples was done but returned negative. Finally, the diagnosis retained was the association of HOA and SNPT.

He was treated with antitubercular therapy for six months according to the national program for the fight against tuberculosis (isoniazid, rifampin, ethambutol, and pyrazinamide for two months (intensive phase), followed by isoniazid and rifampin for four months (continuation phase)).

HOA symptoms had remitted after three months of treatment. Chest CT performed two months after stopping treatment showed regression of the lung cavity (Figure 2(b)). Bone scintigraphy performed six months later showed improvement in scintigraphic abnormalities (Figure 3(b)).

Furthermore, the patient did not present any adverse and unanticipated events during the treatment period.

## 3. Discussion

HOA is a clinicoradiological syndrome, associating an acromelic syndrome (digital clubbing, swelling of extremities, and vasomotor disorders), bone syndrome (periostosis and acro-osteolysis), joint syndrome, and skin syndrome. There are two forms of HOA, the primary and the secondary form [1]. HOA is most often secondary to pulmonary neoplasms (80% of cases) [2]. The absence of similar cases in the family and the lack of skin syndrome in our patient do not argue in favor of a primary HOA, especially with the existence of pulmonary involvement, keeping in mind the genetic variability of primary HOA and the nonspecificity of skin syndrome [1]. The favorable clinical and scintigraphy evolution under antitubercular therapy reinforced us in the eventuality that the secondary HOA is more probable than the primary HOA.

Association of HOA and pulmonary tuberculosis is rarely reported, especially in Africa where the prevalence of tuberculosis is high [3, 5]. Our patient presented a HOA associated with SNPT.

The diagnosis of pulmonary tuberculosis is made with the help of chest X-rays, AFB smear microscopy, and sputum culture, but the diagnostic delay is more or less important [6]. Molecular diagnosis has significantly reduced tuberculosis diagnosis delays. However, molecular methods performed significantly worse in the case of negative microscopy. Therefore, the cost and unavailability of these methods in our country limit their use. For example, Xpert MTB/RIF is a multigeneration automated molecular assay that detects tuberculosis and rifampicin resistance within 2 hours [6, 7].

Although the SNPT accounts 30–65% of all pulmonary tuberculosis cases, its diagnosis is challenging in underdeveloped countries [8]. Diagnosis of SNPT is based on negative smears (at least two sputum samples), chest X-ray findings consistent with tuberculosis, and failure to respond to broad-spectrum antibiotics for several days [6]. If SNPT is suspected, the International Standard for Tuberculosis Care (3^rd^ edition) recommends Xpert MTB/RIF testing and/or sputum culture [9]. Our patient cannot benefit from Xpert MTB/RIF due to unavailability.

Pulmonary tuberculosis associated with HOA is often radiologically extensive and cavitary. Cavitary pulmonary tuberculosis (45% of cases) has an elective apical localization [4].

In our patient, an apical segmentectomy with histopathological examination would have made it possible to make a diagnosis of certainty of tuberculosis or malignancy, but we did not opt for this invasive method because the patient had no neoplastic risk factors or alteration of the general condition.

In the past decade, only two clinical observations have described an association between HOA and tuberculosis. One case of hypertrophic osteoarthropathy associated with pulmonary tuberculosis was reported in 2011 [4]. Another case was reported in 2015, concerning a patient suffering from pulmonary tuberculosis complicated by HAO [10]. These two observations depict two different cases: the first is a HAO discovered concomitant with pulmonary tuberculosis and the second is known pulmonary tuberculosis placed on treatment, complicated by HOA. In our patient, the HOA revealed pulmonary tuberculosis.

Periostosis is the “signature” of HOA but remains neither necessary nor sufficient for the diagnosis. It can be detected by simple radiography and bone scintigraphy; the latter method is more sensitive. In 1226 patients with lung cancer, 55 (4.5%) had periostosis on bone scintigraphy and only ten (0.8%) had clubbing and joint pain [11].

In addition to diagnostic interest, bone scintigraphy can also be used to evaluate therapy response, as periostosis can resolve after etiological treatment [12]. In our patient, the bone scintigraphy confirmed the relationship between SNPT and HOA, as the periostosis resolved after antitubercular treatment.

## 4. Conclusions

In conclusion, HOA associated with pulmonary tuberculosis is rare, in particular with SNMP, which is a difficult diagnosis. The search for tuberculosis in front of any HOA seems to be justified in our epidemiological context. Bone scintigraphy, by its sensitivity to detect periostosis, makes it possible to assess the response to etiological therapy of HOA.

## Data Availability

No data were used to support this study.
